# Prevalence and associations of asymptomatic left ventricular systolic dysfunction in Lebanese patients with type 2 diabetes mellitus

**DOI:** 10.1371/journal.pone.0304801

**Published:** 2024-09-18

**Authors:** Elsy hany El Tawil, Rita Saliby, Ramy Halabi, Joey El Khoury, Serge Assaf, Mira Hamdan, Gilbert Abou Nader, Elizabeth Abou Jaoude

**Affiliations:** 1 School of Medicine and Medical Sciences, Holy Spirit University Of Kaslik, Kaslik, Lebanon; 2 Faculty of Medicine and Medical Sciences, Department of Internal Medicine, Endocrinology Division, University of Balamand, Koura, Lebanon; 3 Department of Internal Medicine, Endocrinology Division, Gilbert and Rose-Marie Chagoury School of Medicine, Lebanese American University, Jbeil, Lebanon; University of Campania Luigi Vanvitelli: Universita degli Studi della Campania Luigi Vanvitelli, ITALY

## Abstract

**Background:**

Diabetes Mellitus is a prevalent disease with a growing impact on individuals worldwide. Evaluating the prevalence of subclinical left ventricular dysfunction and understanding its associations with microvascular complications, uncontrolled glycemia, diabetes duration, and patient age is crucial. Our aim is to determine the utility of screening for this condition.

**Methods:**

We conducted a retrospective cohort study involving 159 asymptomatic individuals with type 2 diabetes. Bivariate analysis was employed to assess potential factors and their associations with subclinical left ventricular dysfunction. Patients with a history of cardiac disease or interventions were excluded.

**Results:**

The average age of our sample was 61.5 years. Almost half of the patients exhibited an HbA1c exceeding 7% (50.3%), and approximately half had an ejection fraction (EF) of less than 55% (50.9%). In the bivariate analysis, a notable difference in microvascular diabetic complications was observed among different EF groups. Specifically, nephropathy (62%), neuropathy (57.5%), and retinopathy (74.4%) were significantly more prevalent among patients with an EF < 55%. We also identified a significant age difference between groups, with a higher mean diabetes duration (14.1 ± 7.7 years) in the lower EF group. Notably, 63.7% of patients with an HbA1c exceeding 7% exhibited an EF < 55%. Older patients were associated with a lower EF, with an adjusted odds ratio (aOR) of 0.94. An HbA1c of 7% or less was linked to a higher likelihood of an EF > 55%.

**Conclusion:**

We established a correlation between subclinical left ventricular systolic dysfunction and microvascular complications. However, further extensive prospective research is necessary to deepen our understanding of these associations and their clinical implications.

## Introduction

We are familiar with and have encountered the recent Coronavirus Disease of 2019 (COVID-19) pandemic, but what about the increasing prevalence of non-communicable diseases? One such non-infectious disease that is a significant global concern is diabetes, with a rapidly rising incidence projected to impact 783.2 million individuals by 2045, as indicated by data from various studies [1]. Diabetes can be caused by either insulin resistance or insulin insufficiency, often associated with beta cell dysfunction. The disease is characterized by two distinct entities: type 1, which is autoimmune in nature, and type 2, accounting for 90–95% of cases [2]. Both type 1 and type 2 diabetes are further complicated by macrovascular diseases such as Cardiovascular Disease (CVD), Cerebrovascular Disease, and Peripheral Vascular Disease (PAD), as well as microvascular diseases including diabetic kidney disease, diabetic retinopathy, and diabetic neuropathy. These complications are the primary contributors to morbidity and mortality in diabetic patients, with CVD being the most prevalent cause of death, accounting for 44% of deaths in Type 1 diabetes and 52% of deaths in Type 2 diabetes [3, 4].

The development of microvascular and macrovascular issues differs between type 1 and type 2 diabetes. In type 1 diabetes, there is no presence of oxidative stress, fibrosis, or inflammation [5]. In type 1 diabetes, the incidence of heart failure is lower compared to type 2 diabetes and is often preceded by hypertension [6].

The Framingham study demonstrated a heightened risk, ranging from two to five times, of cardiovascular diseases and complications, particularly left ventricular systolic dysfunction and heart failure, in individuals with diabetes compared to those without diabetes [7].

While ischemia is the underlying cause of cardiovascular complications across all populations, it is noteworthy that many diabetic patients with subclinical left ventricular systolic dysfunction do not exhibit signs of coronary artery disease [8]. Some studies propose that, in diabetic patients, particularly those with type 2 diabetes, the progression to heart failure may be driven more by hyperinsulinemia rather than hyperglycemia. This is in contrast to type 1 diabetes, where hyperinsulinemia is not typically present. This progression can occur even in the absence of common risk factors such as obesity, hypertension, dyslipidemia, and aging [9].

Enhanced physical and financial quality of life for individuals with type 2 diabetes hinges on the early detection of cardiac dysfunction in these patients [10].

Due to the lack of sufficient studies on asymptomatic left ventricular systolic dysfunction in diabetics, particularly in the Arab world and Lebanon, we selected this topic for our research. We aimed to study left ventricular systolic dysfunction in asymptomatic diabetic patients and factors like diabetes duration,microvascular complications,smoking,age,hypertension,dyslipidemia, and obesity on cardiac dysfunction,emphasizing early screening with cardiac ultrasound. Our hypothesis suggests a potential relationship between the onset of subclinical left ventricular systolic dysfunction and the development of the aforementioned issues.

## Methodology

A retrospective cohort study design was utilized to anonymously access data on type 2 asymptomatic diabetic patients on September 29, 2023, from an endocrinologist’s clinic archive. This access was granted after obtaining IRB ethical approval in December 2022 until January 2023, with the understanding that the data will be destroyed once the legal retention period expires. Inclusion criteria for the study included factors such as age, gender, duration of diabetes, Body Mass Index (BMI), personal history of hypertension and dyslipidemia, smoking habits, familial history of coronary artery disease, use of SGLT-2 inhibitors, latest Hemoglobin A1c (HbA1c) levels, presence of microvascular complications like diabetic nephropathy, neuropathy, and retinopathy, as well as Ejection Fraction (EF) levels. Patients with a positive personal history of heart failure or coronary artery disease, those who had undergone coronary artery bypass surgery or cardiovascular interventions such as Percutaneous Coronary Intervention (PCI), or individuals receiving heart failure treatments like Sacubitril/Valsartan (Entresto®) were excluded from the study. All patients underwent a cardiac ultrasound using M-MODE or SIMPSON METHOD for ejection fraction measurement.

### Statistical analysis

The data was initially collected on an Excel sheet and subsequently analyzed using SPSS v.26. Only 12 missing values were encountered, specifically related to diabetic retinopathy. Descriptive statistics utilized means and standard deviations for continuous variables, while frequencies and percentages were employed for reporting categorical variables. Two continuous variables, HbA1c and EF levels, were dichotomized as follows: 7% or less and higher than 7% for HbA1c, and above and below 55% for EF (based on the American College of Cardiology Foundation stage B definition of Asymptomatic left ventricular dysfunction). Bivariate analysis was conducted to compare ejection fraction with sample characteristics. For categorical variables, the Chi-square test (χ2) was used to identify significant differences, while independent Student’s t-test was employed for continuous variables. A p-value of ≤0.05 was considered indicative of a significant statistical difference, with a confidence interval of 95%. Finally, a logistic binary regression was performed with EF as the dependent variable and predictors being the variables with a p-value <0.2 in the bivariate analysis.

## Results

**Fig 1:**
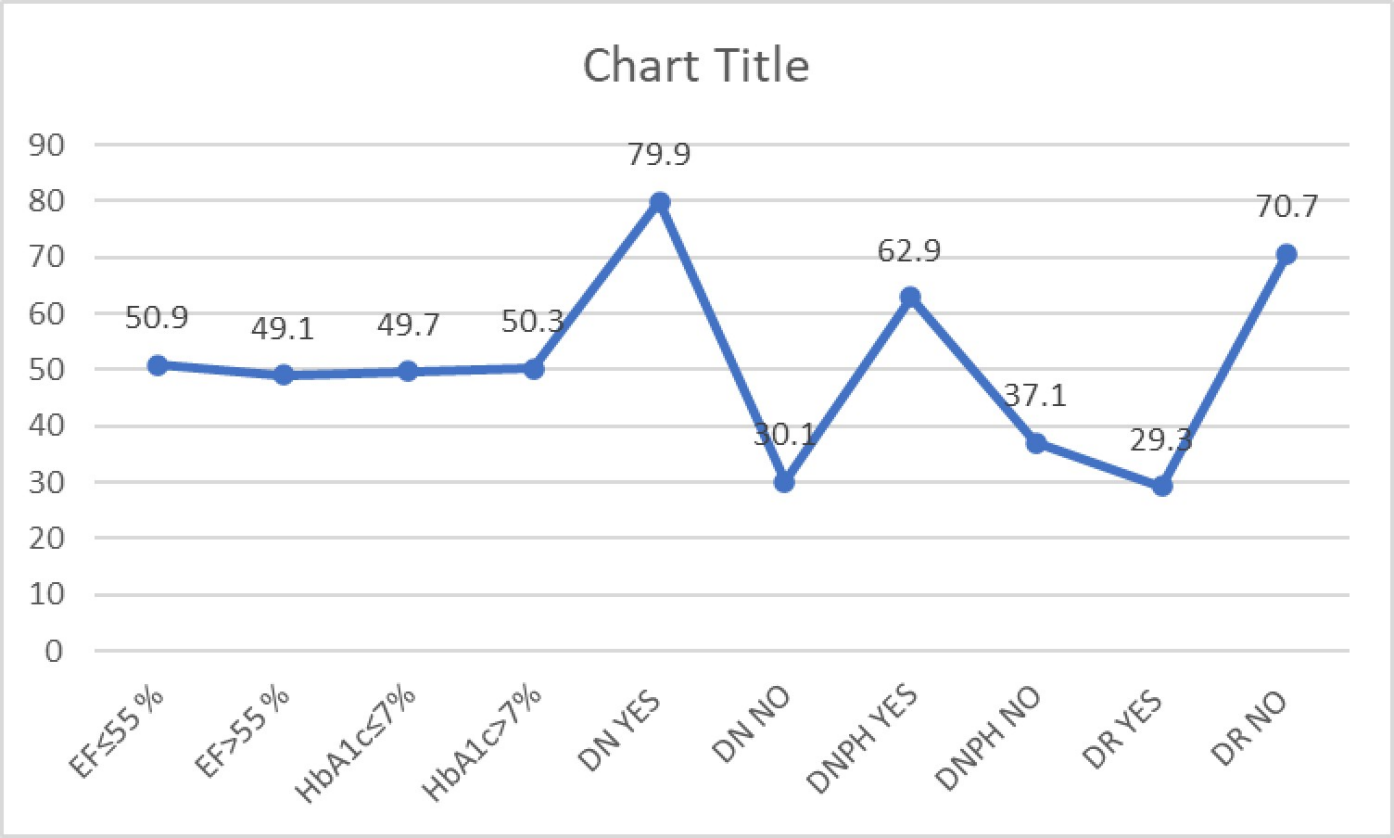
Percentages of left ventricular systolic dysfunction, hbA1c levels and microvascular complications in diabetic patients. EF:ejection fraction; HbA1c:hemoglobin A1c; DN:diabetic neuropathy; DNPH:diabetic nephropathy, DR:diabetic retinopathy.

A total of 159 patients’ data were collected and analyzed. The mean age of our sample was 61.5 years old. Descriptive statistics “Table 1”, “Fig 1” revealed that approximately half of the patients had an HbA1c level above 7% (50.3%) and an EF below 55% (50.9%). In the bivariate analysis “Table 2”, a significant difference was observed when comparing the presence of microvascular diabetic complications among different EF groups. Specifically, nephropathy (62%), neuropathy (57.5%), and retinopathy (74.4%) were significantly more prevalent among patients with an EF below 55%. Additionally, a significant difference was noted in the mean age of our groups “Table 2”. Furthermore, the mean duration of diabetes (in years) was higher (14.1 +/- 7.7) (p = 0.03) in the lower EF group. Moreover, 63.7% of patients with an HbA1c level above 7% had an EF below 55% (p = 0.001).

**Table 1:** Descriptive statistics.

Variable N = 159		N (%)
Age (years), mean: 61.5 (SD: ±12.0)		
Diabetes duration (years), mean: 12.9 (SD: ±7.7)		
Gender	Male	103 (64.8)
	Female	56 (5.2)
Hypertension	Yes	131 (82.4)
	No	28 (17.6)
Dyslipidemia	Yes	149 (93.7)
	No	10 (6.3)
Smoking	Yes	81 (50.9)
	No	65 (40.9)
	Stopped	13 (8.2)
Obese	Yes	93 (58.5)
	Overweight	25 (15.7)
	No	41 (25.8)
SGLT2 inhibitor treatment	Yes	120 (75.5)
	No	39 (24.5)
Diabetic neuropathy	Yes	127 (79.9)
	No	32 (30.1)
Diabetic nephropathy	Yes	100 (62.9)
	No	59 (37.1)
Diabetic retinopathy	Yes	43 (29.3_
	No	104 (70.7)
Family history of CAD	Yes	107 (67.3)
	No	52 (32.7)
HbA1c	7% or less	79 (49.7)
	Higher than 7%	80 (50.3)
EF%	55% and lower	81 (50.9)
	Higher than 55%	78 (49.1)

Sodium/Glucose Cotransporter 2 (SGLT2); CAD:coronary artery disease;HbA1c:hemoglobin A1c;EF, Ejection Fraction

**Table 2:** Bivariate analysis taking EF% as a dependent variable (binary and ENTRESTO excluded).

Variables		EF groups		P-value
		55% and lower	Higher than 55%	
Age (Years)		65 +/- 12.1	57.3 +/- 10.4	0.001
Diabetes duration (Years)		14.1 +/- 7.7	11.5 +/- 7.6	0.03
Gender	Male	54 (52.4)	49 (47.6)	0.61
	Female	27 (48.2)	29 (51.8)	
Smoking	Yes	38 (46.9)	43 (53.1)	0.24
	No	38 (58.5)	27 (41.5)	
	Stopped	5 (38.5)	8 (61.5)	
Hypertension	Yes	72 (55)	59 (45)	0.02
	No	9 (32.1)	19 (67.9)	
Dyslipidemia	Yes	78 (52.3)	71 (47.7)	0.17
	No	3 (30)	7 (70)	
Obesity	Obese	48 (51.6)	45 (48.4)	0.94
	Overweight	13 (52)	12 (48)	
	No	20 (48.8)	21 (51.2)	
SGLT-2 inhibitors	Yes	64 (53.3)	56 (46.7)	0.29
	No	17 (43.6)	22 (56.4)	
Diabetic nephropathy	Yes	62 (62)	38 (38)	0.001
	No	19 (32.2)	40 (67.8)	
Diabetic neuropathy	Yes	73 (57.5)	54 (42.5)	0.001
	No	8 (25)	24 (75)	
Diabetic retinopathy	Yes	32 (74.4)	11 (25.6)	0.001
	No	44 (42.3)	60 (57.7)	
Family history of CAD	Yes	56 (52.3)	51 (47.7)	0.61
	No	25 (48.1)	27 (51.9)	
HbA1c	7% or less	30 (38)	49 (62)	0.001
	Higher than 7%	51 (63.7)	29 (36.3)	

Sodium/Glucose Cotransporter 2 (SGLT2); CAD:coronary artery disease;HbA1c:hemoglobin A1c;EF, Ejection Fraction

T test was used for the bivariate analysis for age and diabetes duration and the chi square test was used for the rest of the variables mentioned in the table above.

In the multivariate analysis, logistic regression was conducted with EF as the dependent variable and the group with EF 55% and lower as the reference group. Predictors were selected based on the previous bivariate analysis with a p-value of less than 0.2. The logistic regression model was significant with a pseudo-R2 of 0.312 “Table 3”. Older patients were associated with a lower EF with an adjusted odds ratio (aOR) of 0.94. Additionally, having an HbA1c level of 7% or less was linked to a higher likelihood of having an EF above 55% (aOR: 2.8).

**Table 3:** Logistic regression.

Variable	aOR	p-value	95% Confidence interval	
EF			Lower	Upper
(55% and lower* / Higher than 55%)				
Age	0.94	0.006	0.90	0.98
Diabetes duration	1.006	0.81	0.95	1.06
Hypertension	0.8	0.7	9.25	2.4
(No*/ Yes)				
Dyslipidemia	1.6	0.58	0.29	8.8
(No*/ Yes)				
Diabetic nephropathy	0.57	0.22	0.23	1.4
(No*/ Yes)				
Diabetic neuropathy	0.49	0.18	0.17	1.39
(No*/ Yes)				
Diabetic retinopathy	0.59	0.29	0.22	1.5
(No*/ Yes)				
HBA1c	2.8	0.01	1.27	6.2
(7% or less / Higher than 7%)				

*: Reference group.

Pseudo-R^2^ Nagelkerke = 31.2%

Sodium/Glucose Cotransporter 2 (SGLT2); CAD:coronary artery disease;HbA1c:hemoglobin A1c;EF, Ejection Fraction

## Discussion

### Pathogenesis

There is still much to be understood about the pathophysiology of left ventricular systolic dysfunction and its progression to heart failure in diabetic patients. In an autopsy conducted on four patients afflicted with diabetic nephropathy and heart failure, Rubler et al. found no indication of coronary artery disease (CAD). They postulated that the observed myocardial fibrosis and hypertrophy were contributory factors to the advancement of heart failure in diabetes [11, 12]. In 1977, Regan reaffirmed the aforementioned assertions through an autopsy conducted on eleven uncomplicated diabetic patients, of whom nine exhibited no evidence of coronary artery disease (CAD) and succumbed to heart failure. The examination revealed a buildup of perivascular intermuscular fibrosis alongside elevated levels of triglycerides. These discoveries hint at an extravascular mechanism underlying diabetic cardiomyopathy [13].

Studies also suggest that insulin resistance and hyperglycemia play a significant role in this process, leading to increased concentrations of free fatty acids and growth factors that disrupt the balance in cardiomyocytes through mitochondrial dysfunction [14–16]. Oxidative stress, triggered by reactive oxygen species, can result in abnormal gene expression and cardiomyocyte fibrosis, hypertrophy and death [17]. All of these are still speculative and need to be thoroughly investigated.

Upon dividing the study population based on ejection fraction (EF) levels, it was observed that over 50% had a decreased EF (<55%). The presence of microvascular complications in diabetes (nephropathy, neuropathy, retinopathy) revealed that a majority of patients with these comorbidities had an EF <55%. This supports the theory of similarities in the pathogenesis of microvascular and macrovascular (cardiovascular) complications [15, 18, 19].

### Diabetic retinopathy and subclinical ventricular dysfunction

In diabetic patients with retinopathy, 74.4% had an EF <55%, aligning with previous studies. Some researchers utilized global longitudinal strain measurement as a prognostic factor for left ventricular systolic dysfunction, while others employed stress tests to exclude coronary artery disease patients. These studies demonstrated a correlation between microvascular complications and asymptomatic left ventricular dysfunction in diabetics, emphasizing the importance of screening for prevention [17]. The pathogenesis of diabetic retinopathy is believed to be linked to hyperglycemia-induced oxidative stress, leading to vascular disruption in the eyes [20].

### Diabetic nephropathy and subclinical ventricular dysfunction

Similarly, hyperglycemia and hypertension are implicated as root causes of diabetic nephropathy progression. Patients with diabetic nephropathy and neuropathy were predominantly found in the lower EF group, indicating their predictive role in cardiovascular disease prevention in diabetic patients. Many observations postulate that insulin is responsible for the upregulation of the sodium-hydrogen exchanger isoform 1 Sodium/Hydrogen Exchanger Isoform 1 (NHE1) in the heart and Sodium-Hydrogen Exchanger Isoform 3 (NHE3 in the kidneys) which when both overly stimulated cause respectively: cardiac hypertrophy, dysfunction and ensuing heart failure, plus fluid retention and nephropathy [21, 22].

This also proves the predictor role these microvascular complications have in cardiovascular disease prevention in patients living with diabetes.

### HbA1c and subclinical ventricular dysfunction

In addition, 63.7% of the patients with an HbA1c of more than 7% had an EF < 55% while the majority of the patients with an HbA1c of < 7% were among the group with an EF of more than 55%.This strengthens the already proven results in the United kingdom prospective diabetes study which showed that an increase in HbA1c and poor glycemic control are behind the higher probability of Cardio-Vascular (CV) complications and heart failure [23]. These findings along with the link between uncontrolled HbA1c and left ventricular systolic dysfunction formerly established, demonstrate as well the importance of follow-up and control of glycemic levels in patients with diabetes.

As for age and diabetes duration, the previously confirmed malefactors were also found to be significantly different between our two clusters.

### Limitations

Our study has several limitations. Firstly, there is a selection bias as patients were only sourced from a single location, which may affect the generalizability of our findings. Additionally, cardiac ultrasonography was performed by multiple cardiologists. Due to financial constraints, we were unable to conduct stress tests to properly exclude symptomatic patients or those with confirmed Coronary Artery Disease (CAD). Furthermore, we did not include Brain Natriuretic Peptide(BNP) and strain measurements to adequately assess ventricular dysfunction. This limitation impedes the complete confirmation of causality between left ventricular systolic dysfunction and microvascular complications. To address this gap, future research should consider conducting a prospective cohort study to establish a causal relationship between microvascular complications and left ventricular dysfunction.

## Conclusion

To the best of our knowledge, this study represents the first of its kind in Lebanon to investigate the Prevalence and associations of Left ventricular systolic dysfunction in asymptomatic diabetic patients. Our findings revealed an association between microvascular issues and early, asymptomatic left ventricular systolic dysfunction, although further in-depth and prospective research is warranted. Despite the study’s limitations, it has provided a basis for linking microvascular and cardiovascular problems. This knowledge should be expanded upon in future cohort studies, which will delve deeper into the etiology of this condition in relation to diabetes mellitus and explore potential prophylactic screening methods using a larger sample size while addressing our study’s weaknesses.

## Supporting information

S1 Data (XLSX)

## List of abbreviations

COVID: Corona Virus Disease of 2019
CVD: Cardiovascular Disease
PAD: Cerebrovascular Disease, Peripheral Vascular Disease
BMI: Body Mass Index
SGLT2: Sodium/Glucose Cotransporter 2
HbA1c: Hemoglobin A1c
EF: Ejection Fraction
PCI: Percutaneous Coronary Intervention
M-Mode: Motion Mode
NHE1/3: Sodium/Hydrogen Exchanger Isoform 1/3
CV: Cardio-Vascular
CAD: Coronary Artery Disease
BNP: Brain Natriuretic Peptide
